# A Unique Complication of Pigtail Pleural Catheter Placement

**DOI:** 10.7759/cureus.69754

**Published:** 2024-09-19

**Authors:** Elizabeth M Sullivan, John D Cambron, Brook Danboise

**Affiliations:** 1 Emergency Medicine, Christus Health/Texas A&M University School of Medicine, Corpus Christi, USA

**Keywords:** chest tube, lung penetration, pigtail catheter placement, pigtail complication, thoracostomy tube

## Abstract

Pigtail pleural catheters are becoming more popular because they are a less painful and equally efficacious alternative to the traditional large-bore thoracostomy tube. This report describes the case of a 48-year-old male whose deteriorating condition required the placement of a pigtail pleural catheter for treatment of parapneumonic pleural effusion. He later developed significant complications related to the procedure.

## Introduction

An alternative to the traditional large-bore thoracostomy tubes, pleural catheters, also called pigtail catheters, are small-caliber (<14 French) chest tubes most often utilized in pneumothorax and pleural effusions [1]. Rather than performing the surgical thoracostomy that larger-bore tubes require, pigtail catheters allow placement via the Seldinger method [1]. This is considered a less painful and invasive method for managing pneumothorax and pleural effusion [1,2]. Other benefits that have been noted are decreased cost, reduced length of stay, fewer ambulatory restrictions, and decreased risk of hemorrhage in anticoagulated patients. Placement of these pigtail chest tubes has become the preferred method and will continue to be so for managing the aforementioned pathologies [1,2]. Because of their widespread use, educating providers about the risks associated with placing these catheters is imperative.

## Case presentation

**Figure 1:**
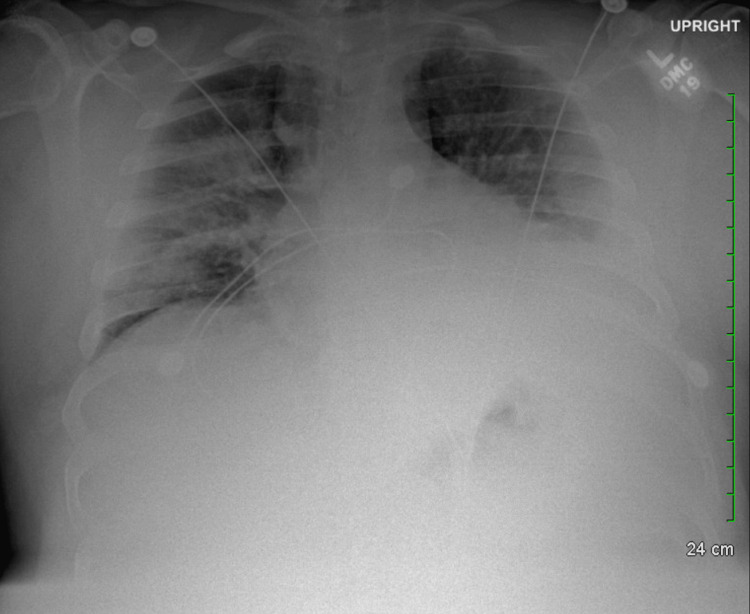
Initial chest X-ray in emergency department

**Figure 2:**
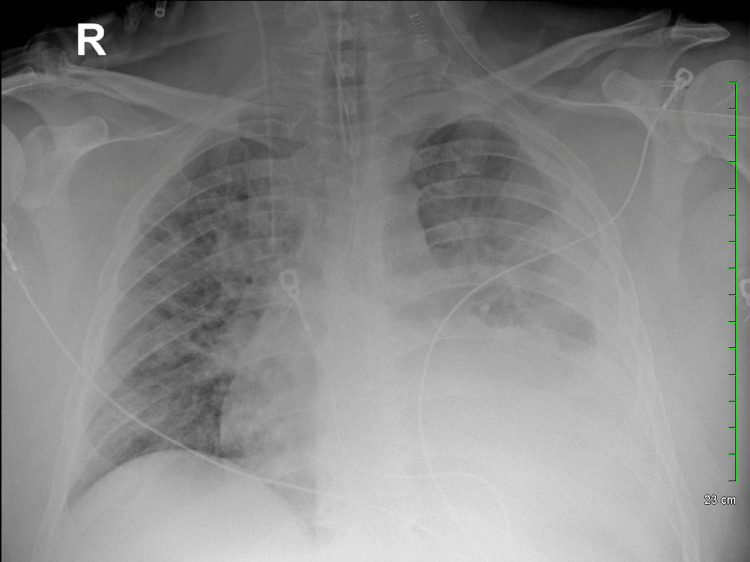
Worsening pleural effusion visualized on X-ray confirming successful endotracheal intubation

A 48-year-old male with a history of hypertension and polysubstance abuse presented to our emergency department (ED) with a chief complaint of shortness of breath. He had approximately five days of shortness of breath, productive cough, and nasal congestion. Upon arrival at the ED, he was sitting in a tripod position with moderate respiratory distress, and his O2 saturation was 92% on room air. He also had mild tachycardia at a rate of 100 and was hypertensive at 201/97. The physical exam was remarkable for crackles worse in the left lower lobe. Laboratory evaluation revealed a leukocytosis at 37.8 with a leftward shift, mild hyponatremia at 128, and an acute kidney injury with a creatinine of 1.9. The patient also had a lactic acidosis of 6.0 on arrival. A single-view chest X-ray obtained in the ED revealed a left-sided opacification vs. pleural effusion (Figure 1). Interventions in the ED included 30 mL/kg intravenous crystalloid bolus, ceftriaxone, and vancomycin for broad-spectrum antibiotic coverage. The patient was admitted to the hospital med-surg floor for severe sepsis secondary to pneumonia, requiring supplemental oxygen. The patient was hemodynamically stable at admission, requiring only 4 L/minute supplementary oxygen via nasal cannula. After the initial admission conversation between the ED provider and hospitalist, the patient became progressively more agitated and restless, requiring increased oxygen. The patient was upgraded to the intensive care unit (ICU) and was subsequently intubated for acute hypoxic respiratory failure. The post-intubation film showed advancing multifocal infiltrates (Figure 2).

The patient’s management and workup were expanded to include a computed tomography (CT) scan of the chest/abdomen/pelvis, and antibiotics were broadened to cefepime, vancomycin, and metronidazole. The CT revealed bilateral multifocal pneumonia with a large left pleural effusion and possible developing empyema. Due to progressively worsening oxygenation and respiratory acidosis despite ventilatory changes, a needle thoracentesis was performed in the ICU overnight. Approximately 200 mL of purulent material was evacuated on thoracentesis. This provided brief stabilization of the patient for the remainder of the first night. Investigation into the patient’s pulmonary etiology resumed the following morning, day 2 of the patient’s hospitalization. This included bronchoscopy, which yielded no significant findings, and bedside ultrasound, which revealed loculations within the left pleural effusion. Therefore, a bedside pigtail catheter was placed into the left thorax via the usual Seldinger technique without complications or concerns. Unfortunately, there was minimal output from this pleural catheter, and intrapleural thrombolytics were given to break up the suspected empyema loculations. Multicenter Intrapleural Sepsis Trial (MIST II) was not followed in this case at the direction of the attending intensivist. Despite these efforts, the patient’s vasopressor requirements continued to increase along with worsening renal function, requiring emergent hemodialysis. An echocardiogram was also obtained to evaluate for infectious endocarditis because of the patient’s history of intravenous drug use. The interpretation was limited secondary to the patient’s body habitus and could not assess for vegetation; however, it revealed an ejection fraction of 55%.

**Figure 3:**
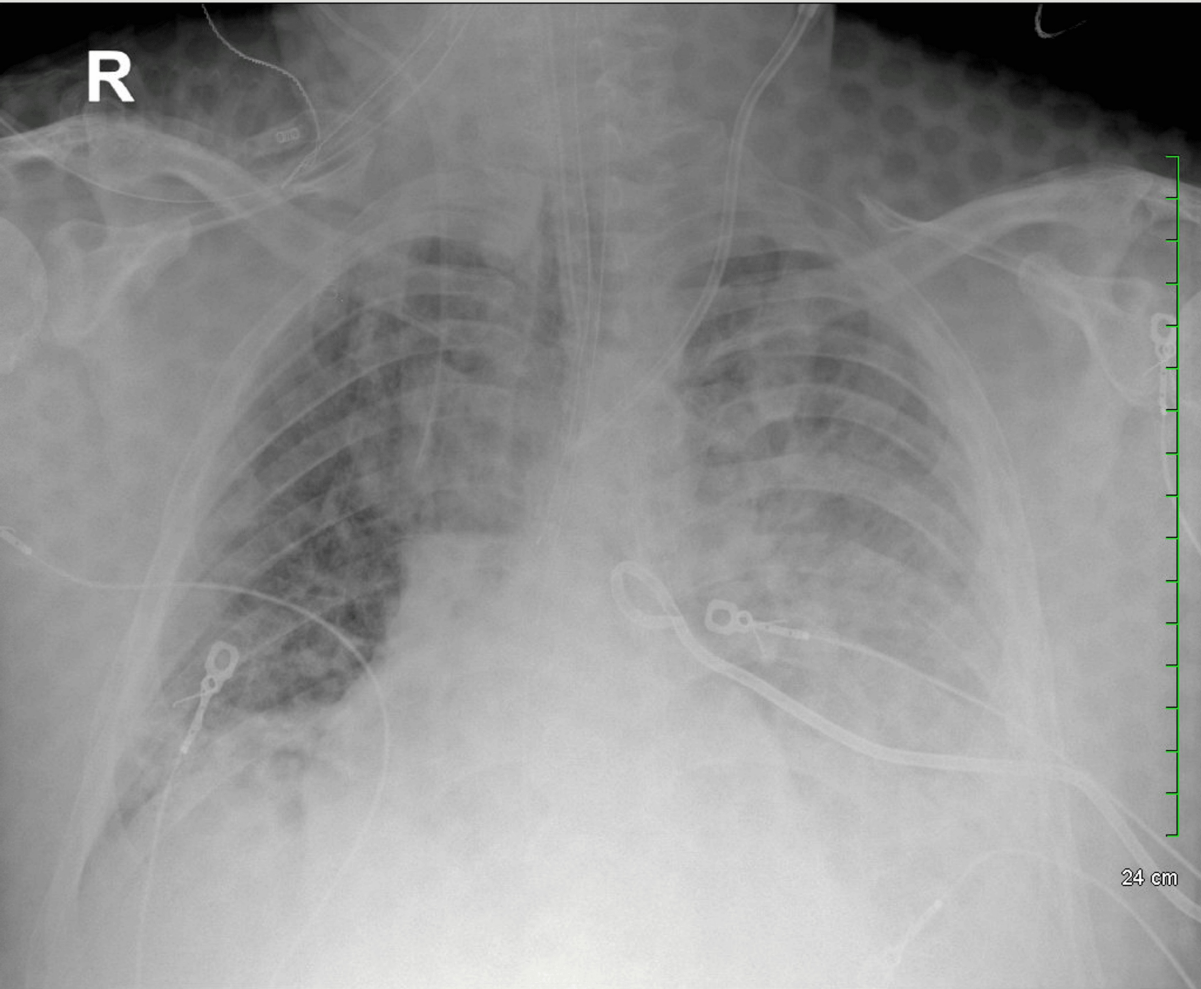
Status post left pleural catheter placement and hemodialysis catheter placement

The clinical course continued to worsen on day 3 of hospitalization, now requiring both norepinephrine as well as vasopressin. Additionally, the patient had become oliguric and was now receiving daily dialysis. A total of two rounds of intrapleural thrombolytics were given with minimal to no output from the pleural catheter. Plain film imaging revealed stable left-sided effusion (Figure 3).

**Figure 4:**
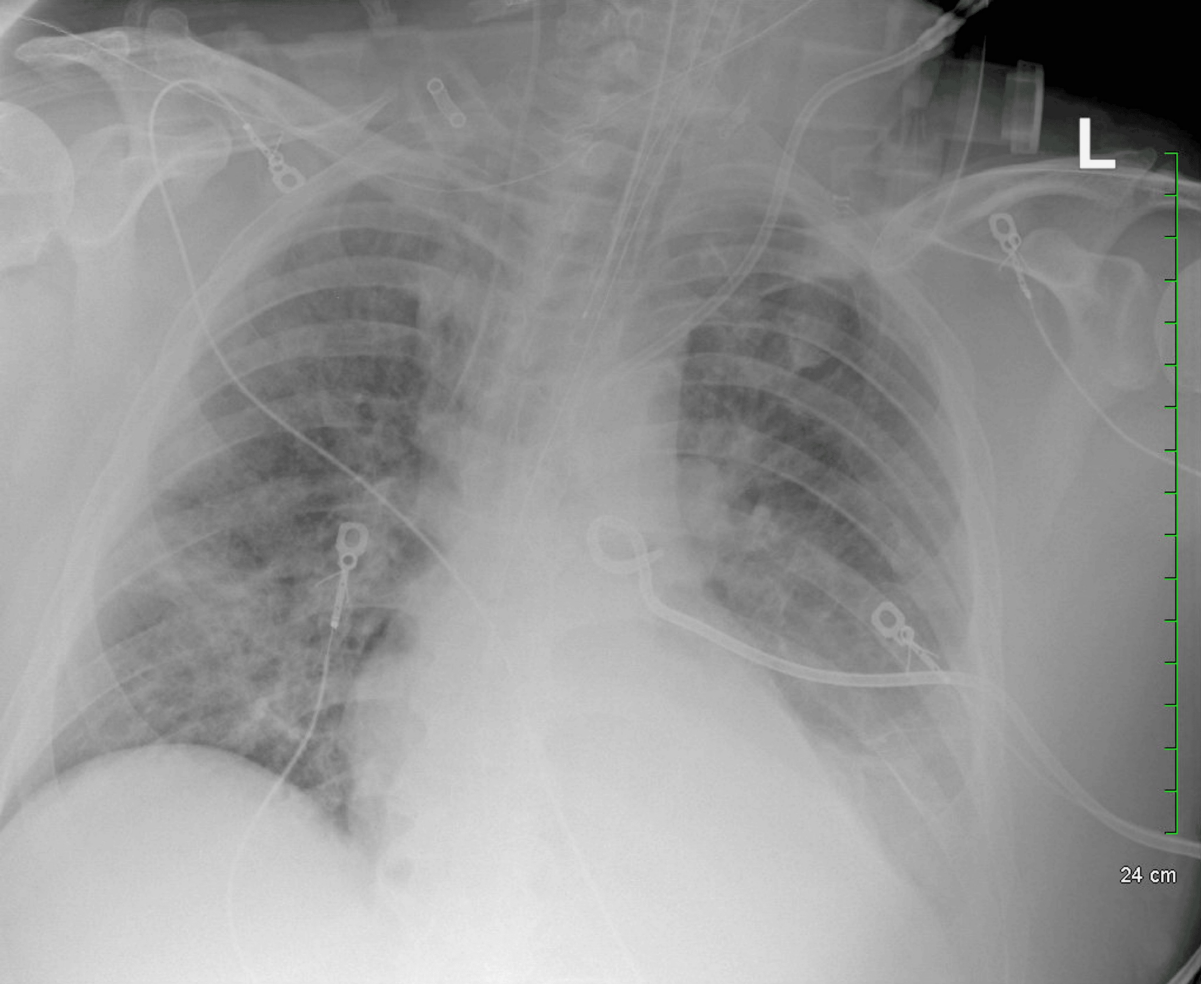
Day 4 chest X-ray, improving infiltrates and left-sided effusion

Although the patient remained in serious condition, he appeared to stabilize during days 4 through 7. Vasopressors were weaned off, and ventilatory requirements improved. Daily chest X-rays indicated improvement of the left-sided effusion and multifocal pneumonia (Figure 4). The patient’s blood cultures revealed *Streptococcus pneumoniae* bacteremia. Pleural fluid from the initial thoracentesis and respiratory culture from the bronchoscopy demonstrated no growth on culture and were negative for acid-fast bacilli.

**Figure 5:**
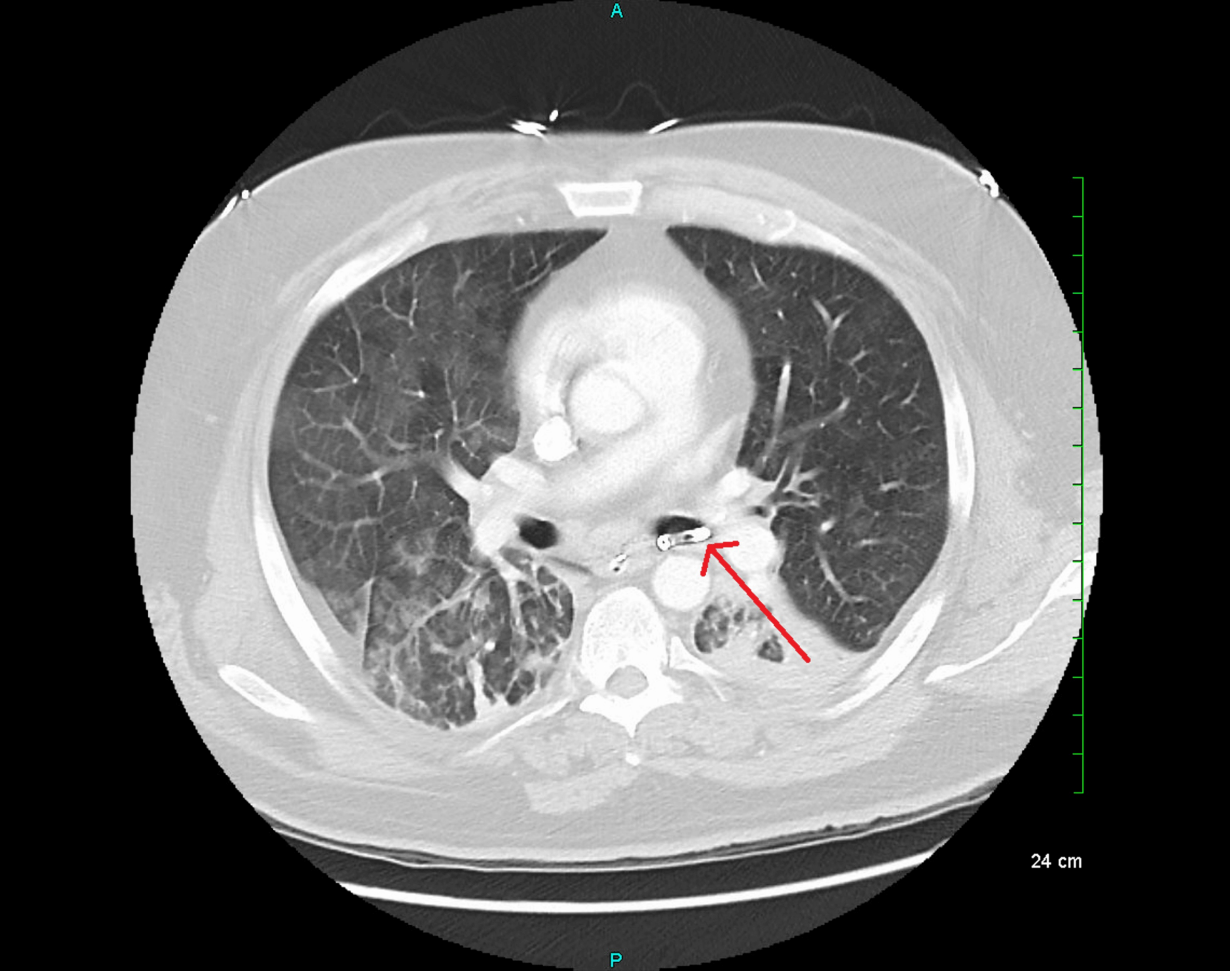
CT angiography chest (axial view) on day 7 with a pleural catheter in the left mainstem bronchus

**Figure 6:**
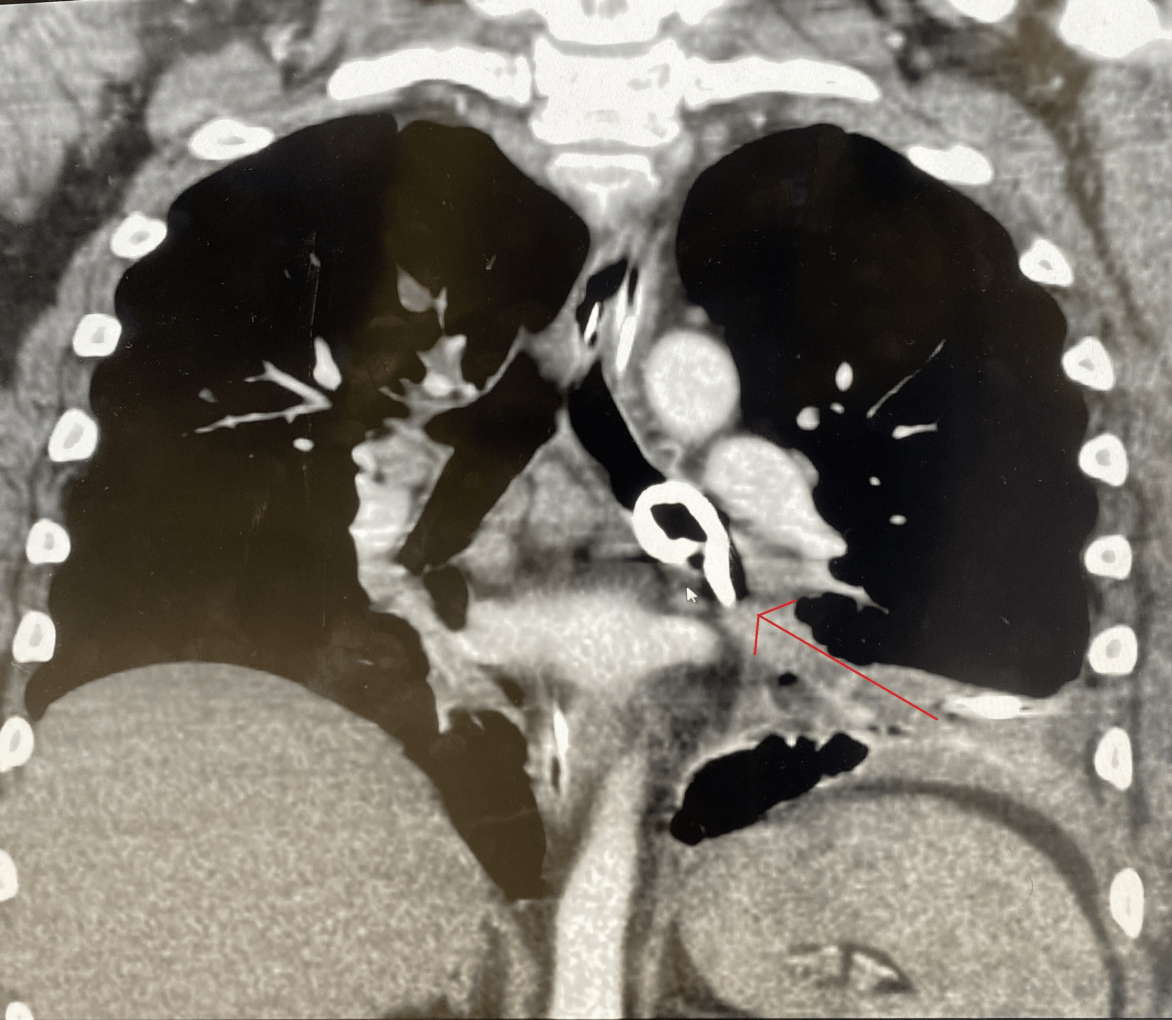
Day 7 CT angiography (coronal view) showing the pleural catheter in the left mainstem bronchus

**Figure 7:**
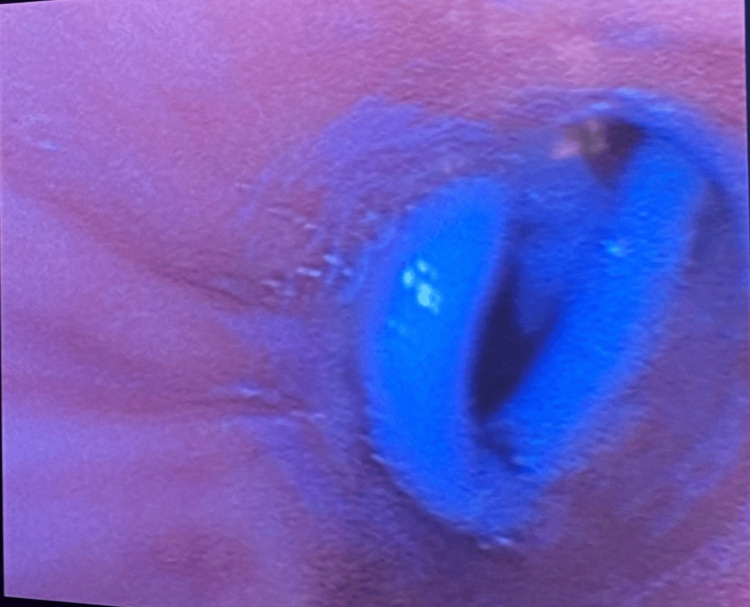
Bronchoscopy of the left main stem bronchus visualizing pleural catheter

**Figure 8:**
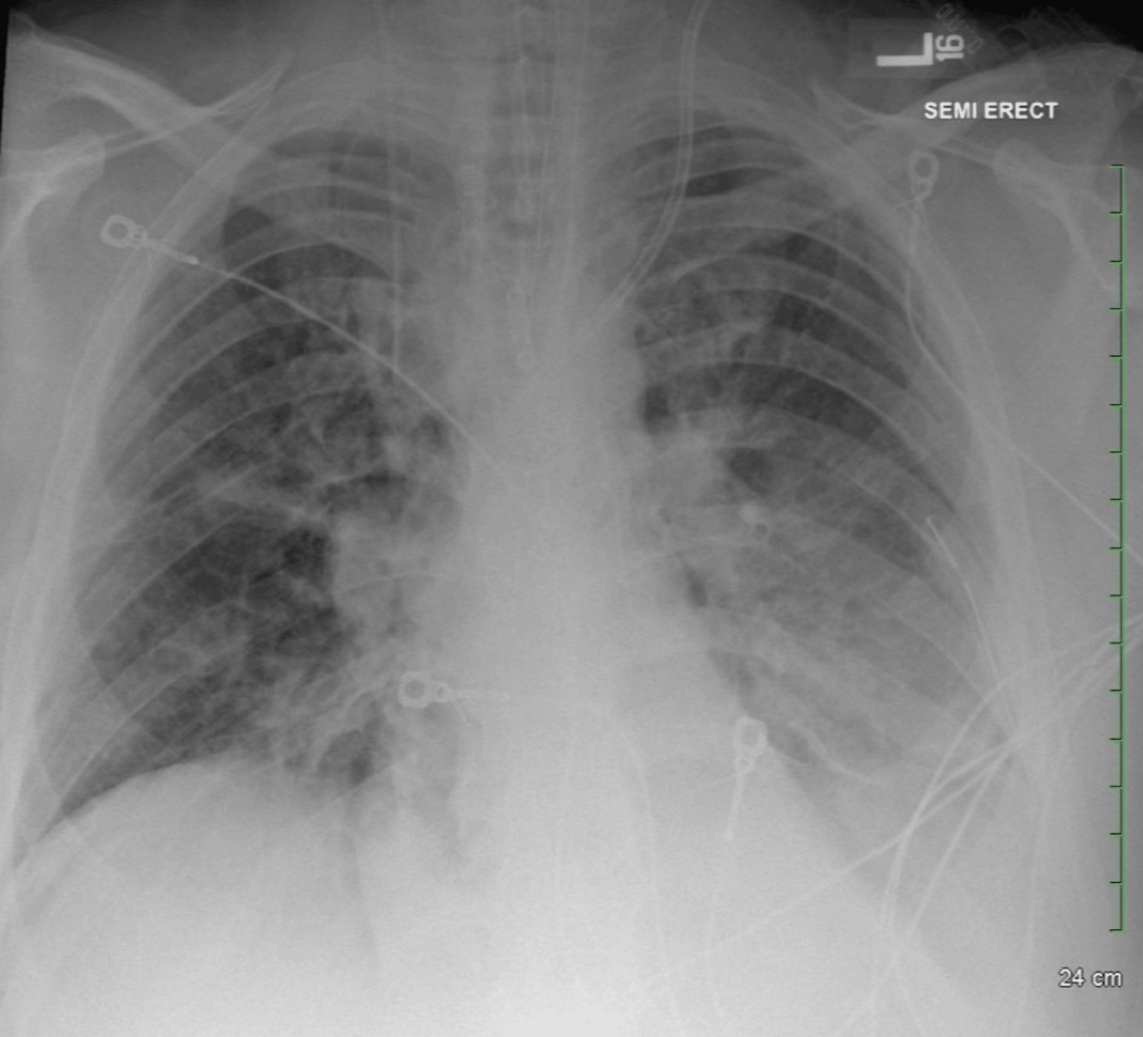
Chest X-ray following pigtail removal and surgical chest tube insertion

On day 7, the patient developed worsening metabolic acidosis and leukocytosis and began requiring vasopressor support again. CT angiography (CTA) of the chest was obtained to evaluate potential pulmonary embolism (Figure 5 and Figure 6). The radiology interpretation revealed the left pleural drainage catheter entering the bronchial tree with its pigtail in the left mainstem bronchus, multifocal pneumonia, small left pleural effusion, and no pulmonary embolism. A bronchoscopy was performed to confirm this imaging finding and indicated that the pigtail catheter was, in fact, in the left mainstem bronchus (Figure 7). Cardiothoracic surgery was consulted, and the cardiothoracic surgeon removed the pigtail catheter at the bedside. A surgical chest tube was placed into the left chest wall immediately following the removal of the pigtail catheter (Figure 8).

## Discussion

Our case report describes a potential risk associated with placing a pigtail catheter at the patient’s bedside via the Seldinger technique. Pigtail catheters have become more commonly utilized over the last several years as an effective and less invasive procedure than the traditional large-bore chest tubes. Like large-bore chest tubes, pigtail catheters are used to evacuate air or fluid from the thoracic cavity [1,2].

A pigtail chest tube placed via the Seldinger technique is less invasive, traumatic, and damaging to nearby structures than the traditional large-bore chest tubes. It has a smaller region of tissue involvement, resulting in better pain control and less scarring. Some challenges include additional procedure steps, more equipment, and increased difficulty confirming placement [1-6].

There are certainly benefits to this procedure when compared to large-bore chest tubes. These include reduction in pain, lower cost, reduced length of stay, fewer ambulatory return restrictions, and decreased risk of hemorrhage in anticoagulated patients. Additionally, pigtail catheters have been shown to be just as effective as large-bore catheters in treating traumatic pneumothorax [2-5]. Pigtail catheters have also been utilized in malignant effusions, but the jury is still out on their effectiveness in comparison to the large bore chest tubes. However, these smaller-bore catheters have a higher risk of acute kinking and blockage and, at times, are thought to have a diminished ability to drain particularly viscous or large volumes of fluids [3].

Complications of chest tubes include infection, damage to nearby structures, kinking/blockage, or, rarely, the introduction of air into the systemic circulation causing cerebral air emboli [2-3,6]. It is more common to encounter intercostal vessel or lung lacerations with traditional large chest tubes [7]. With the smaller pigtail catheters, one cannot physically palpate one’s location; therefore, malpositioning and inappropriate placement are more commonly noted [3]. There have been very few documented incidences of lung penetration with pigtail catheters, but there has been one report by Saqib et al. where a chest tube was placed in the intraparenchymal lung tissue [2].

To our knowledge, there have been no cases of pigtail catheters traversing through to the lung’s bronchial tree. We utilized the Cook Wayne Pneumothorax Catheter Set and Tray (Cook Medical, Bloomington, IN), which includes a pigtail straightening obturator. Our catheter, placed via the Seldinger technique, went through an area of consolidative tissue surrounded by empyema and traversed to the left mainstem bronchus. The empyema’s location was confirmed on ultrasound prior to the procedure. During placement, there was no indication of malpositioning, as no resistance was met while performing the appropriate technique. The procedure overall went smoothly. Unfortunately, this malpositioning was not noted until much later in the patient’s hospitalization due to the limitations of placement confirmation. While we realize that pigtail catheters provide significant benefits, we also must acknowledge that these devices require accurate placement and carry a non-negligible risk of malpositioning during placement.

## Conclusions

Pigtail pleural catheters are being used with increasing frequency in our EDs. As emergency physicians, we need to recognize the potential complications associated with pigtail catheter placement and how to address and avoid them. Unfortunately, we are unable to fully quantify the morbidity of this inappropriately placed catheter, given that our patient was in critical condition at the time of placement. He did show signs of improvement initially after catheter insertion despite malposition. The standard method for confirming placement is a chest plain film, which, in this case, revealed adequate placement for several days. We hope this case report will aid others in preventing and detecting similar complications in the future.
